# Redundant publications in surgery: a threat to patient safety?

**DOI:** 10.1186/1754-9493-2-6

**Published:** 2008-03-19

**Authors:** Philip F Stahel, Pierre-Alain Clavien, Wade R Smith, Ernest E Moore

**Affiliations:** 1 Department of Orthopaedic Surgery, Denver Health Medical Center, University of Colorado School of Medicine, 777 Bannock Street, Denver, CO 80204, USA; 2 Department of Visceral Surgery, University Hospital Zurich, Ramistr. 100, CH-8091 Zurich, Switzerland; 3 Department of Surgery, Denver Health Medical Center, University of Colorado, School of Medicine, 777 Bannock Street, Denver, CO 80204, USA

## Outlining the problem

A redundant publication is a manuscript which fundamentally presents results from the same study in more than one original paper. This term is synonymous with a "dual" or "duplicate" publication of identical data (so-called "self plagiarism") and with the disaggregated presentation of identical data in multiple publications derived from the same study (so-called "salami slicing science"), published by the same author or group. Hereby, the content of redundant papers may overlap in part or completely, such that the main findings of an original study are published in multiple papers in different electronic or print journals.

Redundant publications in biomedical journals are considered unethical for the following reasons [1-3]:

• "Inflation" of the available peer-reviewed literature.

• Skewing of evidence-based medicine when readers erroneously assume to be confronted with reports from independent studies.

• Distortion of available scientific data by unjustified overestimation of a therapeutic effect in systematic meta-analyses.

• Increased, unnecessary workload for editors and peer reviewers, leading to a backlog of "true" original articles in the publication process.

• Cost-ineffective use of resources, waste of journal space, and waste of readers' time by reading republished material considered to be original work.

• Distortion of the purpose of biomedical journals as being the source of new information.

• Potential infringement of international copyright law.

Duplicative scientific publications are being uncovered and reported at an alarming rate in the peer-reviewed surgical literature [4,5]. In the field of general surgery, the screening of 660 original articles in three major peer-reviewed surgical journals revealed that 14% of original papers had published redundant data [6]. In orthopedic surgery, the prevalence of redundant original publications was found to be 3% to 8% [7,8]. Impressively, the screening of a yearly volume of *The Journal of Bone and Joint Surgery *(British and American volumes), which is considered the most prestigious journal in the field of orthopedics, revealed that one in 13 original articles were either duplicate or fragmented publications [8]. These numbers emphasize the prevalent problem of redundant original publications in the peer-reviewed surgical literature.

## Is there a necessity for redundant publications?

There are certain types of articles which may provide a benefit for the scientific community if published in duplicate versions. This includes the repeated publication of official clinical guidelines in order to reach a broader, interdisciplinary readership by being published in the corresponding organs of different professional societies. Furthermore, the translation of published articles from non-English to English language journals and vice versa may support the global spread of important scientific information. Finally, an editor may deliberately choose to invite a replica of a significant non-original article as a secondary publication in a different journal. Under any of the above-mentioned circumstances, the secondary publication must state in a footnote on the title page that the manuscript has been previously published in whole or in part, and must reference the original source of publication in the footnote and in the cited bibliography. Such secondary publications must also be accompanied by a written copyright release statement by the original publisher, in order to prevent a potential infringement of copyright law.

## What characterizes a fraudulent redundant publication?

Redundant scientific publications are considered highly unethical when published in a covert, deceptive fashion [2,9,10]. The main motivation for an author to publish unauthorized redundant work is the increased publication record based on an apparent, but fictitious, scientific productivity. This artificial boosting of curricula vitae is done for the benefit of undeserved academic promotions, grant funding opportunities, and prestige in the scientific community.

A consensus statement by the editors-in-chief of 23 major surgical journals defined fraudulent work related to redundant publications as [11]:

• "Falsely certifying that the submitted work is original and has not been submitted to, or accepted by, another journal."

• "Falsification of any item on the copyright form."

According to the criteria established by the *International Committee of Medical Journal Editors *(ICMJE), any submitting author must make a full statement to the editor about all submissions and previous publications of the same or very similar work [1]. Any such publication must be referred to and referenced in the new paper, and copies of such material should be included with the submitted paper. Redundant publications which do not adhere to these guidelines are considered scientifically unethical and fraudulent.

## A challenge to patient safety?

Authors who deliberately publish redundant original work in a covert, deceptive fashion have adopted manifold strategies to divert the public from perceiving the dual publications as fraudulent. For example, authors of covert redundant papers usually change the order of authorship on duplicate papers, change the corresponding author, and submit their manuscripts to multiple journals and in different languages [12-17]. Authors also choose to publish secondary papers in non-indexed journals which they can list on their academic CV, but will not appear on a PubMed database search by third parties.

Fraudulent redundant publications may pose a threat to patient safety when the authors' deception strategy includes the falsification and fabrication of data, in order to distract from the redundancy of publication. For example, a recent biomechanical study designed to test spine implants was published as two "original" articles which were based on the identical experimental study [12,13]. Aside from changing the order of authors and the language of the manuscript (German and English), the authors also switched the designation of implants used in the two manuscripts. This led to a secondary manuscript in which the implant claimed to be tested ("locking compression plate"; LCP) no longer reflected the original implant ("limited-contact dynamic compression plate"; LCDCP) which had truly been tested in this biomechanical study. The use of a different implant designation may have been aimed at distracting from the presence of a dual publication by screening of abstracts in online databases, such as PubMed. The secondary paper presented purely fictitious data, based on the intent of diverting from a covert redundant publication. Thus, redundant publications may represent a significant threat to patient safety, particularly if conclusions and clinical recommendations are based on fabricated data [13]. Unauthorized duplicate publications may furthermore challenge patient safety by skewing the evidence-based literature and thus altering the individual physician's clinical decision making, based on available meta-analyses and guidelines, towards an unjustified treatment regimen which may have been published in the literature in multiple versions. Unethical behavior has an unfortunate tendency to not remain isolated. Thus, authors who knowingly and covertly submit duplicate manuscripts must be considered at increased risk for data fraud, inappropriate author listings, and possible human review board violations.

A review on the prevalence of redundant surgical publications appropriately observed that "leading surgical journals have hitherto remained largely silent on this issue" [6]. The *British Journal of Surgery *was noted to be an exception by playing a dominant role in disclosing and retracting fraudulent redundant publications in the field of surgery on a regular basis [5,18,19]. As journal editors, we have the responsibility to uphold the high ethical standards of scientific publishing by taking the necessary precautions to assure our readers that the papers published in our journals reflect "true" science, based on objective data and honest scientific reporting. If an unauthorized redundant publication is discovered in the submission phase, the paper must be rejected. If it is detected after publication, the paper must be retracted and the appropriate boards and institutions should be notified [1,2,20,21].

## Guidelines for an acceptable "dual" publication

As outlined in detail by the consensus statement on "Uniform requirements for manuscripts submitted to biomedical journals" by the *International Committee of Medical Journal Editors *(ICMJE), readers of biomedical journals should be able to trust the primary source to represent original work, unless accompanied by a clear statement that a particular article is being republished by the choice of the author and editor [1]. Unauthorized redundant publications may occasionally result from an individual lack of awareness of the published guidelines and ethical standards of scientific publishing. Therefore, in conclusion, we wish to emphasize the official requirements for an "acceptable secondary publication", as defined by the ICMJE guidelines [1]:

1. "The authors have received approval from the editors of both journals; the editor concerned with secondary publication must have a photocopy, reprint, or manuscript of the primary version".

2. "The priority of the primary publication is respected by a publication interval of at least one week (unless specifically negotiated otherwise by both editors)".

3. "The paper for secondary publication is intended for a different group of readers; an abbreviated version could be sufficient".

4. "The secondary version faithfully reflects the data and interpretations of the primary version".

5. "The footnote on the title page of the secondary version informs readers, peers, and documenting agencies that the paper has been published in whole or in part and states the primary reference. A suitable footnote might read: *This article is based on a study first reported in the [title of journal, with full reference*]. Permission for such secondary publication should be free of charge".

6. "The title of the secondary publication should indicate that it is a secondary publication (complete republication, abridged republication, complete translation, or abridged translation) of a primary publication".

According to the official consensus statement by the *Surgical Journals Editors Group *[11], the following criteria define an acceptable redundant or duplicate publication:

• Prior publication in meeting program abstract booklets and proceedings from scientific meetings. These must be acknowledged and referenced in the final manuscript.

• Expansion of the original database, published in the primary source, by 50% or more. Previous manuscripts reporting the original database must be referenced in the secondary publication.

## Conclusion

Ethical standards in publishing place a high level of self responsibility upon publishers, editors, authors and readers. The scientific/medical community at every level should safeguard the medical literature upon which so many of our therapeutic concepts are based and so many of our patients are treated. The "slippery slope" of self rationalization that leads a physician or scientist to justify unauthorized duplicate publication is the same deceptive impulse that results in false data and unjustified conclusions. Ultimately, what we publish and read may end up impacting the care and outcome of individuals who present to us for relief of disease and suffering. There is no line on any academic curriculum vitae worth a human life. On behalf of our patients, we must remain vigilant and educate ourselves, our colleagues, and our students on the high ethical standards of scientific publishing.

## Competing interests

The author(s) declare that they have no competing interests.

## Authors' contributions

All authors contributed equally to the design and writing of this editorial. All authors read and approved the final manuscript.
